# Duodenal Fluid Analysis as a Rewarding Approach to Detect Low-Abundance Mutations in Biliopancreatic Cancers

**DOI:** 10.3390/ijms25158436

**Published:** 2024-08-02

**Authors:** Francesca Tavano, Anna Latiano, Orazio Palmieri, Domenica Gioffreda, Tiziana Latiano, Annamaria Gentile, Matteo Tardio, Tiziana Pia Latiano, Marco Gentile, Fulvia Terracciano, Francesco Perri

**Affiliations:** 1 Division of Gastroenterology and Endoscopy, Fondazione IRCCS “Casa Sollievo della Sofferenza” Hospital, Viale Cappuccini 1, 71013 San Giovanni Rotondo, FG, Italy; 2 Department of Surgery, Fondazione IRCCS “Casa Sollievo della Sofferenza” Hospital, Viale Cappuccini 1, 71013 San Giovanni Rotondo, FG, Italy; 3 Department of Oncology, Fondazione IRCCS “Casa Sollievo della Sofferenza” Hospital, Viale Cappuccini 1, 71013 San Giovanni Rotondo, FG, Italy

**Keywords:** biliopancreatic cancers, liquid biopsy, duodenal fluid, mutation profiling, somatic variants

## Abstract

Diagnosis of biliopancreatic cancers by the available serum tumor markers, imaging, and histopathological tissue specimen examination remains a challenge. Circulating cell-free DNA derived from matched pairs of secretin-stimulated duodenal fluid (DF) and plasma from 10 patients with biliopancreatic diseases and 8 control subjects was analyzed using AmpliSeq™ HD technology for Ion Torrent Next-Generation Sequencing to evaluate the potential of liquid biopsy with DF in biliopancreatic cancers. The median cfDNA concentration was greater in DF-derived than in plasma-derived samples. A total of 13 variants were detected: 11 vs. 1 were exclusive for DF relative to the plasma source, and 1 was shared between the two body fluids. According to the four-tier systems, 10 clinical tier-I–II (76.9%), 1 tier–III (7.7%), and 2 tier–IV (15.4%) variants were identified. Notably, the 11 tier-I-III variants were exclusively found in DF-derived cfDNA from five patients with biliopancreatic cancers, and were detected in seven genes (KRAS, TP53, BRAF, CDKN2A, RNF43, GNAS, and PIK3CA); 82% of the tier-I-III variants had a low abundance, with a VAF < 6%. The mutational profiling of DF seems to be a reliable and promising tool for identifying cancer-associated alterations in malignant cancers of the biliopancreatic tract.

## 1. Introduction

Biliopancreatic cancers, including pancreatic cancer and cholangiocarcinomas (CCAs), are aggressive and are often diagnosed at an advanced stage, resulting in a dismal prognosis. Pancreatic ductal adenocarcinoma (PDAC) is the most prevalent histologic type of pancreatic cancer; PDAC precursors include microscopic lesions represented by pancreatic intraepithelial neoplasia (PanIN) and macroscopic mucinous lesions, namely, intraductal papillary mucinous neoplasm (IPMN) and mucinous cystic neoplasm (MCN), both of which are characterized by low or high dysplasia or by the presence of invasive carcinoma [1,2]. CCAs constitute a diverse group of rare malignancies arising in the biliary tract [3]; CCAs are often indistinguishable from PDAC by standard histopathology because the two have overlapping immunohistochemical profiles, and differentiation between PDAC and CCAs is based mainly on tumor location [4].

The diagnosis of biliopancreatic cancer is based on a combination of medical history and physical examination, serum tumor marker levels (i.e., CA19-9: carbohydrate antigen 19-9; CEA: carcinoembryonic antigen), imaging techniques (i.e., US: ultrasonography; EUS: endoscopic ultrasound; ERCP: endoscopic retrograde cholangiopancreatography; CT: computed tomography; MR: magnetic resonance), and pathological confirmation of tissue specimens. Notably, an increase in serum CA19-9 and CEA may occur in several diseases in the absence of malignancy, limiting the role of these molecules in the diagnosis and monitoring of biliopancreatic cancer [5]. All the available imaging-based techniques also have low sensitivity for cancer precursor lesions and low specificity in differentiating either nonmalignant from malignant lesions or among several inflammatory conditions [6,7]. Moreover, most biliopancreatic cancers are inoperable at diagnosis; thus, surgical specimens are unavailable, and tissue sampling for cytological testing is most often performed with ultrasound (US)-guided percutaneous biopsy, EUS-guided fine-needle aspiration (FNA) and/or fine-needle biopsy (FNB), biopsy forceps, or a biliary brush during ERCP. However, for various reasons, such as inadequate material or low cellularity, such sampling could result in nondiagnostic or indeterminate interpretations [8,9,10].

In this scenario, innovative investigations are needed to improve early diagnosis, treatment selection, disease monitoring, and response rate evaluation. Liquid biopsy, based on the detection of different molecules released by tumors into body fluids, represents a minimally invasive, repeatable, and cost-effective method for the molecular characterization of cancer [11]. Circulating cell-free DNA (cfDNA) refers to short fragments of DNA liberated into almost all body fluids, including blood, as a result of apoptosis, necrosis, and secretion, and is involved in various physiological and pathological phenomena. In cancer patients, the fraction of cfDNA originating from cancer cells is termed circulating tumor DNA (ctDNA) and may encompass the same genetic and epigenetic information as those of the primary tumor from which it originates. Previous studies suggested that the genetic profiling of plasma ctDNA might significantly improve current systems for tumor diagnosis, tumor progression monitoring, targeted therapies, and early-stage detection [12]. However, the search for biomarkers in the blood has several pitfalls, including the very low amounts of biomarkers present in the circulation and the assumed correlation between the local release of cancer-associated molecules and their measurable concentration in the blood [13]. Conversely, investigations on proximal fluids gathered in the direct vicinity of affected organs could reduce the biological hitches of other circulating body fluids. Indeed, as biomarkers are released into the bounded fluidic environment, the proximal fluid contains the highest concentration of DNA, proteins, and exosomes released from normal or injured organs, increasing the sensitivity of detecting biomarkers derived from cancer and high-risk lesions [14]. Studies exploiting the analysis of duodenal/pancreatic fluid (DF) samples have revealed preliminary exciting information on molecular alterations associated with biliopancreatic cancers. Mutant *KRAS* may provide evidence for the presence of PanINs, and *TP53* or multiple *KRAS* mutations have been associated with invasive IPMNs [15,16,17,18]. Specific variations in the *TP53*, *GNAS*, and *SMAD4* genes have emerged as markers of invasive cancer and high-grade dysplasia or for distinguishing adenocarcinoma from IPMN [19,20,21,22]. Several differentially expressed protein markers, microRNAs, and aberrantly methylated genes have also been reported [23,24]. In patients with CCAs, differences in the metabolomics profile of DF samples between malignant and benign biliary diseases have been revealed [25].

This study aimed to evaluate the potential of DF as a new promising throve of information about cancer-associated and transforming mutations in biliopancreatic cancer. For this purpose, circulating cfDNA-based next-generation sequencing (NGS) was performed on secretin-stimulated DFs and matched plasma samples from patients with biliopancreatic cancer and control subjects. We hypothesized that DF would enclose many more changes than those occurring in the bloodstream and that it may serve to elucidate the genetic events underlying the transformation toward an inflammatory or neoplastic process to help physicians improve the detection of malignancy at the molecular level and the clinical management of patient care.

## 2. Results

### 2.1. Diagnosis of Biliopancreatic Diseases in the Study Population

The final diagnosis of biliopancreatic disorders based on imaging and cytological tests of tissue specimens from the study population is summarized in Supplementary Table S1. Histological confirmation of biliopancreatic diseases required repeated tissue sampling attempts in most patients in our study population. Overall, cytological evaluation confirmed the diagnosis in six out of the eight tested patients (one with IPMN-derived PDAC, two with PDAC, one with IPMN, and two with pancreatitis), while two patients with CCAs were fine-needle aspiration cytology-negative.

CA19-9 measurements were available for 15/18 (83.3%) subjects. The median concentrations were 153.9 UI/mL (range 2.2–2374), 196 UI/mL (range 6.8–1092.6), and 5.9 UI/mL (range 0.01–30.1) in samples from patients with malignant diseases (IPMN-derived PDAC, PDAC, and CCAs), benign diseases (IPMN and pancreatitis), and controls, respectively. In addition, the CA19-9 level was greater than the normal value (37 UI/mL) in 67% (4/6) of the patients with malignant disease, 50% (2/4) of the patients with benign disease, and 0% (0/8) of the control subjects.

### 2.2. Circulating Cell-Free DNA in Matched Duodenal Fluid and Plasma Samples

The cfDNA concentration was undetectable by the Qubit fluorometer in the DF from one PDAC patient; in the remaining samples, the median cfDNA concentrations from 1 mL of DF eluted in 25 µL of elution buffer were 976.8 ng/mL (range: 151.4–1375), 456.7 ng/mL (range: 173.8–627), and 576.3 ng/mL (range: 28.4–2500) in samples from malignancies, benign diseases, and controls, respectively; no significant difference in DF-derived cfDNA concentrations emerged between malignant and benign cases (*p* = 0.556) or between malignant or benign diseases and controls (*p* = 0.799 and *p* = 0.777, respectively).

The median cfDNA concentrations from 1 mL of plasma eluted in 25 µL of elution buffer were 72.8 ng/mL (range: 25.5–97), 64.2 ng/mL (range: 9.5–100.1), and 38.3 ng/mL (range: 8.8–285) in samples from patients with malignancies, patients with benign diseases, and controls, respectively. Similarly to DF, no significant difference between malignant and benign diseases (*p* = 0.914) or between malignant or benign diseases and controls (*p* = 0.573 and *p* = 0.808, respectively) was observed.

**Figure 1:**
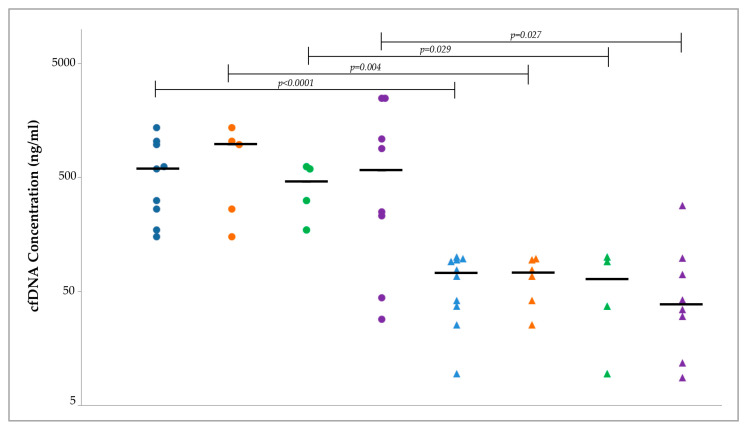
Circulating cell-free DNA (cfDNA) concentration in matched duodenal fluid and plasma samples. Each dot and triangle represent a single cfDNA measurement in duodenal fluid and in plasma, respectively, on log scale (y-axis). Blue dots/triangles: malignant + benign biliopancreatic diseases; orange dots/triangles: malignant biliopancreatic diseases; green dots/triangles: benign biliopancreatic diseases; violet dots/triangles: controls. The median levels in each subgroup are indicated by the horizontal black bars.

Overall, although high interindividual variability was observed, in almost all cases, marked differences between plasma- and DF-derived cfDNA concentrations were detected (Table 1). The distribution of cfDNA concentration in DF and plasma samples across different subgroups of patients with biliopancreatic diseases and controls is represented in Figure 1.

The Wilcoxon signed-rank test showed that the cfDNA concentration in samples from patients with biliopancreatic disease was significantly greater in DF-derived samples than in plasma-derived samples, either when all the samples were analyzed together (598.8 ng/mL vs. 72.8 ng/mL, *p* < 0.0001) or when malignant and benign samples were considered separately (PDAC/CCAs: 976.8 ng/mL vs. 72.8 ng/mL, *p* = 0.004; IPMN/pancreatitis: 456.7 ng/mL vs. 64.2 ng/mL, *p* = 0.029); similarly, the cfDNA concentration in samples from control subjects was greater in DF-derived samples than in plasma-derived samples (576.3 ng/mL vs. 38.3 ng/mL, *p* = 0.027).

No significant correlation was revealed between cfDNA concentrations in matched DF and plasma samples (Spearman’s correlation coefficient r = −0.276, *p* = 0.282). In addition, the correlation analysis revealed that the concentrations of DF-cfDNA did not correlate with the CA19-9 levels either when all samples were analyzed (r = −0.156, *p* = 0.594) or when only samples with CA19-9 levels > 37 U/L (n = 5) were considered (n = 5; r = 0.6, *p* = 0.350); similar results were obtained when the correlation between the concentrations of plasma-cfDNA and the CA19-9 levels was tested in all the samples (r = 0.354, *p* = 0.196) and in those with CA19-9 levels > 37 U/L (n = 6; r = 0.714, *p* = 0.136).

### 2.3. Circulating Tumor DNA Analyses in Matched Duodenal Fluid and Plasma Samples

The ctDNA detection in DF and plasma samples was carried out by targeting *KRAS* gene mutations. A *KRAS* mutation at codon 12 was detected by ddPCR in DF-derived cfDNA samples from 15/18 (83.3%) subjects (3 PDAC patients, 1 CCA patient, 2 IPMN patients, 2 pancreatitis patients, and 7 controls). The mean fractional abundance (FA) value (±standard deviation) was 0.7% (+1.4) in cases and 0.2% (+0.2) in controls. At least one *KRAS* mutation was detected in 6/18 (33.3%) paired plasma-derived cfDNA samples (1 patients with PDAC, 1 patient with IPMN, 1 patient with pancreatitis, and 3 controls), with mean FA values of 5.1% (+16.1) and 0.2% (+0.5) in the cases and controls, respectively.

Overall, high interindividual variability in the FA value was detected. Thus, the mean FA values in DF and plasma samples from control subjects were used to assign the *KRAS* mutational status in each sample (i.e., the *KRAS* mutational status was considered positive in samples whose FA value was greater than the respective mean FA value in controls). Accordingly, the percentages of patients and controls with *KRAS* mutation positivity were 30% (3/10) and 25% (2/8), respectively, in the DF samples and 10% (1/10) and 12.5% (1/8), respectively, in the plasma samples. The concordance of the *KRAS* mutational status (positive/negative) between DF and plasma was 72.2% (13/18): in 12 subjects (1 PDAC patient, 1 CCA patient, 2 IPMN patients, 2 pancreatitis patients, and 6 controls), the *KRAS* mutation was not detected in the cfDNA from either the DF or plasma, while in 1 control subject, the *KRAS* mutation was detected in both biological sources. Among the five samples with discordant *KRAS* mutational status in the two body sources (27.8%), *KRAS* mutations were detected only in the DF (two PDAC patients, one CCA patient, and one control) or plasma (one PDAC patient) samples; notably, the discordance rate was greater in the patients than in the controls (40% vs. 12.5%).

### 2.4. Sequencing Metrics

A comprehensive list of sequencing parameters is provided in Supplementary Table S2. The ctDNA library concentrations ranged from 128 to 10,227 pM (median 676 pM). A median of 654.749 total read fragments per sample was obtained (range 467.764–2.160.021). A median of 99.20% (range 94.16–99.82%) of all reads per sample was successfully mapped to the reference genome hg19: 98.50%, ranging from 94.2% to 99.5% in plasma samples, and 99.5%, ranging from 97.7% to 99.8% in DF samples. The mean depth sequencing coverage values were 5.071–27.211 (median 7.709): 5.071–11.345 (median 7.387) for plasma and 6.578–27.211 (median 8.734) for DF. The median molecular coverage values were 134–1260 (median 691): 375 ranging from 134–853 for plasma and 856 ranging from 331–1260 for DF.

### 2.5. Somatic Variants Identified in ctDNA Derived from Matched Duodenal Fluid and Plasma Samples

After all the filtering and classification steps, a total of 13 somatic variants were detected in our cohort, of which 2 and 12 variants were found in plasma- and DF-derived cfDNA, respectively. Among all the somatic variants, 11 vs. 1 were exclusively found in cfDNA from DFs relative to plasma, while 1 shared variant was found in cfDNA derived from both body fluids. A total of three variants were detected more than once in two or more subjects. A complete list of the variants identified per patient can be found in Supplementary Table S3.

According to the classification of somatic variants into five biological classes, 11 variants were reported as pathogenic/likely pathogenic, and 2 other variants were benign. Notably, almost all the pathogenic/likely pathogenic variants were classified as tiers I-II according to the clinical class system, with the exception of the c.277C>T variant in the *PIK3CA* gene, which is a clinical class III variant. Overall, the distribution of the identified variants in the four-tier systems revealed 10 tier I–II variants (76.9%), 1 tier –III variant (7.7%), and 4 tier IV variants (15.4%). Notably, all the tier I–III variants were exclusive for DF, while among the two tier –IV variants, one was exclusive for plasma, and the other was found in cfDNA obtained from both body sources.

**Figure 2:**
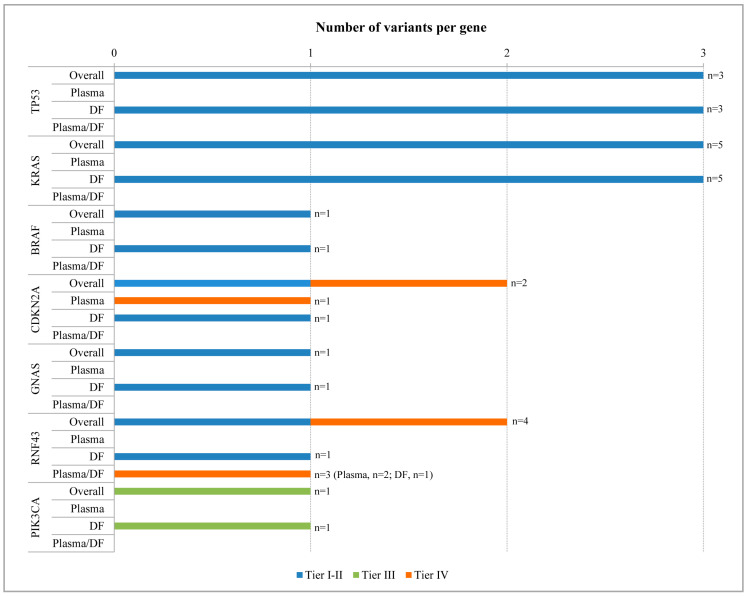
Bar chart of the variants found in cfDNA derived from duodenal fluid (DF) and/or plasma samples. Bars show the total number and the clinical tiers class of variants identified in each gene. The number of samples with mutations is indicated next each bar; for the variant shared between the two body sources, the number of mutated samples is also reported for each sample type.

Per gene distribution of the identified variants throughout the plasma and DF samples of our cohort are shown in Figure 2. The two clinical tier IV variants occurred in the *CDKN2A* and *RNF43* genes, and the latter was altered in both body sources. The 10 identified clinical tier I–II variants were detected in 6 out of the 11 analyzed genes (*TP53*, *KRAS*, *BRAF*, *CDKN2A*, *GNAS*, and *RNF43*) and were all exclusive for DF samples; 30% (3/10) of the clinical tier I–II variants were in the *KRAS* or *TP53* genes, followed by 10% (1/10) in the *BRAF*, *CDKN2A*, *GNAS*, and *RNF43* genes. The clinical tier III variant occurred in the *PIK3CA* gene and was exclusive for DF. No mutations were detected in the *BRCA1*, *BRCA2*, *ERBB2*, or *SMAD4* gene.

**Figure 3:**
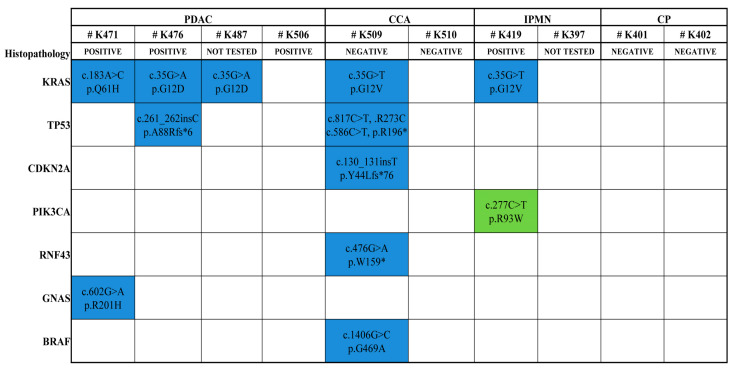
Per-sample occurrence of 11 tier-I–III variants identified across the duodenal fluid-derived cfDNA samples. Patients are displayed accordingly to the disease type, and the results from cytological tests on tissue specimens are shown. Variants in tier I–II are highlighted in blue, and variants in tier III are highlighted in green. * Sample from IPMN-derived PDAC.

Per-sample distribution of the 11 clinical tier I–III variants throughout the DF samples is represented in Figure 3. Variants were detected in 5 out of the 10 patients with biliopancreatic diseases (1 with IPMN-derived PDAC, 2 with PDAC, 1 with CCA, and 1 with IPMN). Notably, two of the identified clinical tier I–II variants were found more than once in two different patients: *KRAS* p.G12D (2 PDAC) and KRAS p.G12V (1 CCA, 1 IPMN). The number of somatic variants detected in patients ranged from 1 to 6: one patient with PDAC had the p.G12D mutation in *KRAS* gene; three patients (1 with IPMN-derived PDAC, 1 with PDAC, and 1 with IPMN) had 2 clinical tier I–II variants in 2 different genes (*KRAS*: p.Q61H and *GNAS*: R201H; *KRAS*: p.G12D and *TP53*: p.A88Rfs*6; *KRAS*: p.G12V and *PIK3CA*: p.R93W); one patient with CCA presented a total of 6 tier I–II variants in 5 different genes (*KRAS*: p.G12V, *TP53*: p.R273C and p.R196*, *CDKN2A*: p.Y44Lfs*76, *BRAF*: p.G469A, RNF43: p.W159*).

### 2.6. Variant Frequency Allele of Variants Identified in Duodenal Fluid Samples

**Figure 4:**
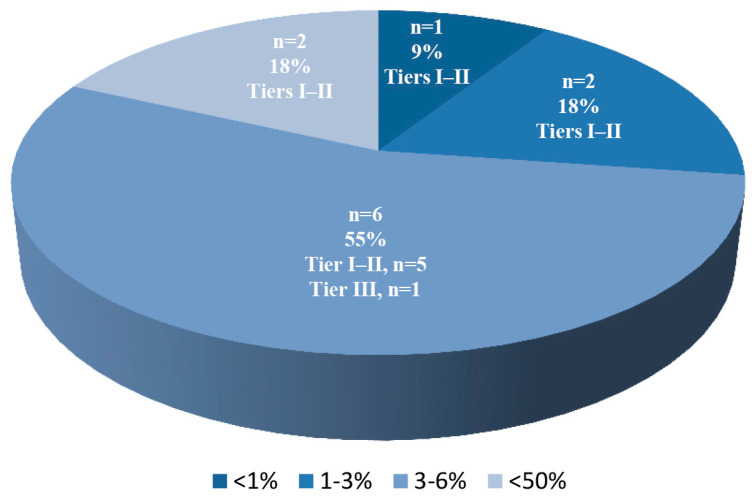
Classification of the 11 tier-I–III variants according to their variant allele frequency values. For each subgroup of variants, the number, the percentage, and the clinical class (tier) is reported.

All the 11 clinical tier I–III variants identified in the DF samples were assigned to four groups according to their VAF values: <1%, 1–3%, 3–6%, and <50% (Figure 4). Notably, among the two variants found in more than one patient, only the *KRAS* c.35G>A variant had a different VAF in the two subjects where it was identified, with a VAF < 1% and a VAF of 1–3%; thus, in both patients, the value was less than 3%. Overall, nine variants (82%) had a low abundance, with a VAF < 6% (VAF < 1%, one variant; VAF 1–3%, two variants; VAF 3–6%, six variants), while two variants (18%) had a high abundance, with a VAF < 50%. Of the variants with a VAF < 6%, 89% (8/9) were classified as tier I–II (VAF < 1%, 1 variants; VAF 1–3%, 2 variants; VAF 3–6%, 5 variants), while 11% (1/9, VAF 3–6%) were in tier III. For class As, for variants with a VAF < 50%, both identified variants were classified as tier I–II.

## 3. Discussion

In this study, the cfDNA derived from matched pairs of DF and plasma samples from 10 patients with biliopancreatic diseases and 8 controls was dissected by targeted sequencing of 591 variants in 11 cancer-associated genes. The purpose was to investigate the potential of DF-derived cfDNA as a source of oncogenic/likely oncogenic alterations associated with biliopancreatic cancer compared to plasma-derived cfDNA. The hypothesis is that, in light of the challenges inherent to traditional biopsies, the evaluation of the potential of detecting cancer with a biofluid proximal to the pancreas and biliary tract could offer a new valuable complementary or alternative way to the conventional approach and break new ground closer to implementing molecular diagnosis into the current standards of care for patients affected by biliopancreatic diseases.

Accurate diagnosis is essential for providing optimal treatment to patients affected by diseases of the biliopancreatic tract, and pathological confirmation of tissue specimens represents an important tool during the diagnostic process. Overall, repeated tissue sampling occurred in most patients within our study population. According to the European Federation of Societies for Ultrasound in Medicine and Biology guidelines [26], US-guided biopsy was at first attempted in patient pancreatic masses suspicious for PDAC. In patients with negative biopsy results and with a high clinical suspicion of malignancy, and when the percutaneous route is not feasible or has failed to provide an adequate diagnosis, the EUS-FNA was considered [27]. For CCA patients, in line with the published literature, the sensitivity of bile duct cytology was unsatisfactory [28,29,30].

In our cohort, we reported a greater median concentration of cfDNA extracted from DF than from plasma samples; the difference was statistically significant when the entire study population was considered and persisted either when the subsets including only patients with malignant or benign diseases were taken into account or when control subjects were compared for median cfDNA concentrations in the two body sources. However, from the evaluation of the median concentration of cfDNA between patients (overall and malignant/benign) and controls and between patients with malignant and benign diseases, no significant differences emerged in either the DF or plasma samples. In addition, cfDNA levels in DF did not correlate with those observed in plasma samples, underlining the potential of DF as a valuable source of cfDNA and, in turn, of its ctDNA component, which is useful for dissecting the genomic landscape of biliopancreatic diseases. Furthermore, the low correlation coefficient between cfDNA concentrations in DFs and matching plasma samples highlighted the complementary nature of the two body fluids as sources of cfDNA. Similarly, no significant correlations emerged between the concentrations of DF- and plasma-derived cfDNA and the serum levels of CA19-9, the latter of which were above the upper normal limits in 67% and 50% of patients with malignant (PDAC and CCAs) and benign (IPMN and pancreatitis) biliopancreatic diseases, respectively. In line with the data reported in the literature, CA 19-9 serum levels did not differentiate between malignant and benign biliopancreatic diseases [31], and the absence of a correlation between the cfDNA concentrations and CA19-9 levels accentuated the usefulness of both liquid biopsy and conventional serum markers as measurable molecular indicators of biliopancreatic diseases.

Herein, we used digital PCR-based techniques to measure ctDNA in plasma- and DF-derived cfDNA. The percentage of detectable ctDNA based on *KRAS* mutations was approximately two and a half times greater in DF samples than in plasma samples. However, high interindividual variability in the FA values was found in both body sources; hence, the mean FA values in plasma and DF samples from control subjects were used to assign positivity to the *KRAS* mutational status in each sample. In this way, the positivity rate of *KRAS* mutation status was greater in DF than in plasma samples in both cases and controls, with cases showing a greater discordance between *KRAS* mutational status in plasma and DF. These data highlight the role of DF as a nonblood source of ctDNA. Furthermore, as discussed below, since the cancer-associated alterations identified in this study were exclusive for DF, we confirmed that the tested cfDNA fragments from DF samples most likely originated from the tumor. The difference in *KRAS* mutational status between the two body sources might be due, as previously reported for other nonblood biological fluids [32], to a more direct contact of DF with tumors, which determines a greater proportion of ctDNA than plasma, where the concentration of target DNA is often very limited and depends on several factors, or to the potential of proximal body fluid to enable the characterization of a genetically distinct population of tumor cells more reflective of the primary tumor.

Ion Torrent HD technology used herein is based on the use of dual molecular barcoding, which enables the clustering of multiple reads originating from the same template DNA for error correction and the identification of rare genetic alterations (VAF > 0.1%) in the liquid biopsy material [33]. Notably, in our study, low-frequency variants were identified by achieving high-quality sequencing (mean depth sequencing coverage: median value of 7.709; median molecular coverage value: median value of 691) and by using an adequate amount of input cfDNA for library preparation (a total of 10 ng). Notably, all these variants were exclusive for DF, while no relevant mutations were highlighted in plasma-derived cfDNA. Nine out of the identified variants (82%) had a low abundance with a VAF <6%, indicating that the variants were of somatic origin. Recently, a mutation analysis of pancreatic juice, plasma, and tissue samples for the detection of pancreatic cancer has been performed by using different NGS platforms and technologies [34]. In line with our findings, a higher concentration of cfDNA in pancreatic juice than in plasma was reported, and in samples tested using the Ion Torrent™ Oncomine™ cfDNA Assays, mutations were detected more frequently in pancreatic juice than in matched plasma samples. In addition, a good correspondence of genes affected by the mutations emerged between the studies. Overall, the identified clinical tier I–III variants included 7 missense, 2 nonsense, and 2 frameshift mutations and were detected in 7 out of 11 analyzed genes: *KRAS*, *TP53*, *BRAF*, *CDKN2A*, *RNF43*, *GNAS*, and *PIK3CA*.

Several clinical implications have been reported for *KRAS* mutations in human malignancies [35,36,37,38]. In pancreatic cancer patients, the presence of p.G12R or p.G12D mutations has been associated with poorer prognosis [39,40], and clinical evidence indicates the therapeutic significance of exon 2 mutations in patients with advanced disease [41,42,43]. Somatic *TP53* mutations have been reported to be predictive of poor prognosis and unfavorable treatment outcomes in human cancers [44,45]. The R273 is a mutation hotspot with p.R273C and p.R273H enhancing proliferation, invasion, and drug resistance in vitro [46]. Codon 196 is a mutational hotspot found in skin, breast, and colon cancers, and the p.R196*truncating mutation has been reported to promote the proliferation and metastasis of tumor cells [47,48]. The p.A88Rfs*6 mutation is likely to result in premature truncation of the protein and a loss of protein function, but this mutation has not been biochemically characterized so far.

No somatic clinical evidence for the *BRAF* p.G469A and the *CDKN2A* p.Y44Lfs*76 variants in biliopancreatic cancer has been reported to date. However, the former mutation has been described among the non-V600 BRAF mutations as a variant with level B prognostic significance in metastatic colorectal cancer, and a plausible therapeutic significance for this variant in patients with advanced refractory solid tumors and lymphomas has also been reported [49,50,51]. Likewise, a plausible therapeutic significance supporting the resistance to palbociclib in advanced pancreatic or biliary cancers with *CDKN2A* loss or mutation has been described [52]. Inactivating RNF43 mutations have been identified in 9.3% of CCAs and serve as an independent predictor of poor survival [53]. The p.W159* mutation identified in a patient with CCA within the present study appears to play a role in the targeting of the Wnt receptor frizzled for ubiquitination and in the removal of *RNF43* from the cell surface with a consequent increase in Wnt activity [54]. Mutations in the *GNAS* gene at codon 201 (p.R201C and p.R201H) determine the constitutive activation of Gsα and its downstream PKA signaling pathway [55], thus representing promising therapeutic targets for GNAS-mutant tumors. These gain-of-function mutations have been identified primarily in IPMN (approximately 65%), subsequently in adenocarcinoma derived from IPMN, and have been reported to not be mutually exclusive with *KRAS* mutations in pancreatic cancer [56,57]. The *PIK3CA* p.R93W mutation identified in this study lies within the PI3K adaptor-binding region and confers a gain of function on the PIK3CA protein compared to wild-type PIK3CA in endometrial tumor cell culture [58]. Preclinical research has also demonstrated its sensitivity to the akt inhibitor miransertib in a patient-derived xenograft model of endometrial cancer harboring *PIK3CA* (p.D350G; p.R93W) and *PTEN* (p.R130G) mutations [59].

Per-sample distribution of the identified tier-I-III variants throughout PDAC patients was consistent with data on the molecular pathology of PDAC, which is characterized by either a low tumor mutation burden and a high prevalence of *KRAS* mutations followed by mutations in TP53, which are relatively late genetic events in the progression model for pancreatic cancer [60]. Indeed, mutations in the *KRAS* gene have been identified in two of the PDAC patients, one of whom also presented a variant in the *TP53* gene. In one other patient diagnosed with IPMN-derived PDAC, the presence of a *KRAS* mutation coexisted with mutations in the GNAS gene, both of which are known to be associated with IPMN [61,62]. Mutations in *KRAS* and in *PIK3CA* have been identified in one patient with noninvasive IPMN. In one PDAC sample, no mutations have been found; given the positive EUS-FNA cytology findings in this sample, it is plausible that our molecular profiling represents a false-negative result or that the presence of other less common molecular alterations not included in the present investigation, such as DNA damage response and epigenetic modifier mutations, could be assumed in this patient [63]. Conversely, despite the negative cytological results obtained for the two CCA patients included in this investigation, one CCA patient had the highest number of mutations in several genes, i.e., *KRAS*, *TP53*, *CDKN2A*, *RNF43*, and *BRAF*, all of which are relevant and have reported evidence for the impact of targeted therapies on CCAs [64,65].

It must be acknowledged that our study has some limitations. First, although this investigation represents the first attempt to use Ion Torrent HD technology to dissect the molecular profile of liquid biopsies in biliopancreatic cancers, the sample size tested was too small and inadequate to perform further statistical analysis, such as evaluation of the associations with patients’ clinical pathological features, outcomes, and response to treatments, which should be further assessed in a larger patient cohort in future studies. In addition, all the patients and controls were enrolled retrospectively into this study; thus, all the subjects with available peripheral blood and DF samples were included in the present evaluation without any specific inclusion criteria, such as the stage of the disease or a specific treatment regimen. Therefore, a prospective cohort study on patients with early and advanced stages of cancer enrolled at different phases of treatment could be useful to test the potential of low-frequency mutations in DF for improving diagnosis, prognosis and longitudinal monitoring of the biliopancreatic cancers. Second, due to the limited availability and quantity of tissue specimens available for cytological analysis, concordance analysis between tissue and liquid biopsy molecular profiles was not performed. However, in a previous study, ctDNA from peripheral blood was compared with that from tumor biopsy samples in patients with biliopancreatic carcinoma, and a 90% concordance in genetic mutations was reported [66]. Anyhow, future research on patients undergoing surgical resection could ensure enough tissue specimens to test the concordance between mutations detected in liquid biopsies and matched solid tumor samples, and identified candidate mutations that drive biliopancreatic cancer development and progression. Third, even though the ddPCR evaluation of *KRAS* mutations at codon 12 carried out to measure the ctDNA portion within the cfDNA allowed us to confirm the presence of the *KRAS* exon 2 variants detected by NGS, with good agreement between the two methods, all the other somatic variants identified in this study were not validated using other methods.

Altogether, our results highlighted that promising information can be derived from the mutation profiling of DF in malignant diseases of the biliopancreatic tract. The AmpliSeq HD technology for Ion Torrent NGS seems to be a reliable tool for the identification of low-frequency cancer-associated variants exclusive for DF compared to matched plasma samples in our study population. However, given the preliminary nature of our findings, further research aimed at better evaluating the applicability of DF as a proximal body fluid for the molecular dissection of biliopancreatic diseases is needed.

## 4. Materials and Methods

### 4.1. Patient Characteristics

A total of 18 subjects were enrolled at the Division of Gastroenterology of the Fondazione IRCCS “Casa Sollievo della Sofferenza” Hospital, San Giovanni Rotondo, Italy. Of these, 10 patients were diagnosed with biliopancreatic disease (3 with PDAC, 1 with IPMN-derived PDAC, 2 with CCA, 2 with IPMN, and 2 with pancreatitis), while the remaining 8 patients attended the outpatient clinic for digestive complaints and, after a clinical evaluation and US disclosing a normal pancreas and biliary tract, were included in this study as the control group because they had environmental risk factors associated with the development of biliopancreatic cancer (i.e., age ≥ 55 years, high body mass index, smoking habit, alcohol abuse, diabetes status, and a family history of cancer in first-degree relatives). The demographic data and associated risk factors for the development of biliopancreatic disorders in the study population are listed in Table 2.

Diagnostic imaging was performed by US in all patients except for 1 patient with PDAC; EUS was performed in 3 patients (1 with IPMN-derived PDAC and 2 with IPMN), and ERCP was carried out in 5 patients (2 with PDAC, 2 with CCA, and 1 with pancreatitis), while in 2 other patients (1 with PDAC and 1 with pancreatitis), both procedures were used; a total of 7 patients (3 with PDAC, 1 with IPMN-derived PDAC, 2 with CCA, and 1 with pancreatitis) underwent CT, and MR imaging was also performed for 2 patients (1 with IPMN-derived PDAC and 1 with CCA). Pathology confirmation of tissue specimens was performed for 8 out of the 10 patients. In detail, tissue sampling for cytological testing was performed with US-guided percutaneous biopsy in 3 patients, EUS-guided FNA in 3 patients, and bile duct brushing or forceps biopsy during ERCP in 4 patients. None of patients enrolled into this study underwent surgical resection of the pancreas and the biliary tract; thus, the pathological diagnosis on the surgical-block specimens was not executed.

Before enrollment into this study, all subjects signed an informed consent form after the aims of this study had been explained. The Ethics Committee of the Fondazione IRCCS “Casa Sollievo della Sofferenza” Hospital approved this study (Prot. No. 49/CE/2017), which was conducted in accordance with the approved guidelines.

### 4.2. Liquid Biopsy Collection

From each study participant, both the DF and the corresponding blood samples were gathered on the day of the endoscopic procedure. Duodenal fluid secretion was stimulated by intravenous infusion of synthetic secretin (Secrelux, Sanochemia, Germany) at a dose of 1 CU/kg body weight over 1–2 min; approximately 10–12 mL of DF was then collected from the duodenal lumen for ~5 min by suctioning fluid through the echoendoscope channel. Tubes containing DF samples were immediately closed, kept on ice, and transferred to the research laboratory of the hospital, where they were centrifuged at 6000 rpm for 5 min at 4 °C. To avoid repeated freezing cycles, the supernatant was aliquoted and suitably stored at −80 °C until use. Blood samples were collected into cfDNA BCT^®^ (Streck, La Vista, NE, USA) tubes. The plasma was separated within 3 h of collection by centrifugation at 3000 rpm for 10 min at 4 °C, followed by a second centrifugation of the supernatant at 12,000× *g* for 5 min at 4 °C, and then, it was stored at −80 °C until extraction.

### 4.3. Isolation and Quantification of Circulating Cell-Free DNA from Duodenal Fluid and Plasma Samples

Circulating cell-free DNA was isolated from 1 mL of DF and plasma using the QIAamp Circulating Nucleic Acid Kit (Qiagen, Hilden, Germany) according to the manufacturer’s instructions. The cfDNA was eluted in 25 μL of nuclease-free water, quantified on a Tape Station 2200 System with D1000 HS reagents and screen tape (Agilent, Santa Clara, CA, USA) and on a Qubit 3.0 Fluorometer by using a Qubit HS DNA Assay Kit (Thermo Fisher Scientific, Inc., Waltham, MA, USA) according to the manufacturer’s instructions, and the cfDNA was stored at −30 °C until use. The mean value between concentrations expressed in ng/μL obtained at via the Qubit and the Tape Station was used to calculate the volume of cfDNA containing a total of 10 ng necessary for library preparation. A previously described fluorometric method was used to calculate the concentration of cfDNA expressed as ng/mL of DF or plasma, which was compared among different patients [67]. Briefly, the following equation was applied: CP = CExVE/VP, where CP corresponds to the concentration of cfDNA in the DF or plasma (ng/mL), CE corresponds to the concentration of extracted cfDNA (ng/μL), VE corresponds to the elution volume used for the cfDNA extraction (μL), and VP corresponds to the volume of plasma or DF used to extract the cfDNA (ml).

### 4.4. Detection of KRAS Mutational Status

*KRAS* mutations were used as targets to detect circulating tumor DNA (ctDNA). Briefly, droplet digital polymerase chain reaction (ddPCR) analysis using the QX200 Droplet Digital PCR system (Bio-Rad Laboratories, Hercules, CA, USA) was performed to detect *KRAS* mutations at codon 12 [68] by using a probe set specific for each mutant (G12D: dHsaMDV2510596; G12R: dHsaMDV2510590; G12V: dHsaMDV2510592; Bio-Rad Laboratories, Hercules, CA, USA) according to the manufacturer’s instructions. A reaction without a template was used as a negative control.

### 4.5. Next-Generation Sequencing Using Ion AmpliSeq HD Technology

A custom panel was designed with the Ion AmpliSeq Designer (https://www.ampliseq.com) using AmpliSeq™ HD technology (Cat. No. A37645, Thermo Fisher Scientific Inc., USA). The panel consists of 75 amplicons covering 591 cosmic variants in 11 genes associated with biliopancreatic cancer transformation and development: *KRAS* (NM_033360.4), *BRAF* (NM_004333.6), *TP53* (NM_000546.6), *SMAD4* (NM_005359.6), *CDKN2A* (NM_001195132.2), *ERBB2* (NM_004448.4), *PIK3CA* (NM_006218.4), *RNF43* (NM_017763.6), *GNAS* (NM_000516.7), *BRCA1* (NM_007294.4), and *BRCA2* (NM_000059.4).

Next-generation sequencing (NGS) libraries were prepared from up to 10 ng of cfDNA using the AmpliSeq™ HD library kit (Cat. No: A37694, Thermo Fisher Scientific Inc., USA) according to the manufacturer’s instructions. Briefly, target amplification reactions were set up following the procedure for 1-pool DNA primer panels (containing FWD and REV subpools) and using cycling option A reported in the Ion AmpliSeq™ HD Library Kit User Guide (MAN0017392). After combining the PCR products, the amplicons were partially digested with 5 μL of SUPA reagent, and the libraries were amplified with 4 μL of the Ion AmpliSeq HD Dual Barcode Kit (Cat. No: A37695, Thermo Fisher Scientific Inc., USA). Finally, the libraries were purified using Agencourt AMPure XP (Beckman Coulter, Brea, CA, USA) and assessed for quality and quantity on D1000 HS screen tape (Cat. No: 5067-5584) on a Tape Station 2200 (Agilent, USA). Barcoded libraries were pooled by combining 3 μL of each 30 pM library. The Ion 510™/520™/530™ Kit-Chef was used for templating and chip loading on the Ion Chef ™ Instrument (Thermo Fisher Scientific Inc., USA). The Ion 520 chip was used for sequencing up to 12 samples, and the Ion 530 chip was used for up to 24 samples. The sequencing run was scheduled on Torrent Suite for the templating protocol at 200 bp. The Ion 510™/520™/530™ Kit-Chef was used for sequencing runs, and the Ion GeneStudio S5™ system was used for sequencing analysis. For sufficient end-to-end amplicon coverage, we set up 500 flows for each sequencing run.

### 4.6. Data Analysis

The raw data were analyzed using Torrent Suite software (Thermo Fisher Scientific Inc., USA) version 5.12.1 running on the Torrent Server (Thermo Fisher Scientific Inc., USA). The pipeline consisted of signal processing, base calling, quality score assignment, adapter trimming, read alignment to the human genome (GRCh37/hg19), mapping quality control, and molecular coverage analysis. Variant calling was performed using Ion Reporter software version 5.18 (Thermo Fisher Scientific Inc., USA) and the Ion AmpliSeq HD Workflow template for Liquid Biopsy-w2.5-DNA-Single Sample and the predefined filter chains “called variants and controls”. The application of UMIs enabled the grouping of reads into molecular families. Random errors generated during library construction and the sequencing process were removed automatically. Apart from the default filter to report the variants, the called variants were further filtered according to the following rules: variants were assumed to be of somatic origin when the variant allele frequency (VAF) was less than 50% [69], alternative allele coverage > 3, alternative allele ratio > LOD, *p* < 0.05, and only variants that alter the protein sequence (i.e., missense, nonsense, in-frame or frameshift indels and splice variants) were retained. After these filtering steps, the remaining variants were classified by using a two-level approach of biological and clinical impact classes; this entails a first classification of variants into a tumor-independent biological class system and a subsequent tumor type-dependent clinical interpretation of somatic variants. First, we applied the workflow of the Commission for Personalized Medicine (ComPerMed) expert panel developed to streamline and harmonize the classification of somatic variants into the five biological classes of the American College of Medical Genetics and Genomics (ACMG) and the Association for Molecular Pathology (AMP) standards and guidelines (i.e., pathogenic, likely pathogenic, variant of unknown significance [VUS], likely benign, and benign) [70,71]. Then, biologically classified variants were attributed to one of the four tiers according to the tiered classification system proposed by the AMP, American Society of Clinical Oncology (ASCO), and College of American Pathologists (CAP) to categorize somatic sequence variations based on their clinical significance: tier I for variants of strong clinical significance in the tumor type investigated; tier II for variants of potential clinical significance; tier III for variants of unknown clinical significance; and tier IV for benign or likely benign variants [72]. In addition, for somatic variants that did not directly align with the AMP/ASCO/CAP designation as tier I, the standard operating procedure for the classification of variants into five oncogenicity categories (i.e., oncogenic, likely oncogenic, VUS, likely benign, and benign) developed by the Clinical Genome Resource (ClinGen), the Cancer Genomics Consortium (CGC), and the Variant Interpretation for Cancer Consortium (VICC) was used to support the assignment of diagnostic, prognostic, or therapeutic significance to variants classified as likely oncogenic or oncogenic, which might be reassigned from the AMP/ASCO/CAP tier III to tier I–II [73]. Franklin Genoox (https://franklin.genoox.com/), a database that provides phenotypic features and variant-level information based on ACMG/AMP and AMP/ASCO/CAP guidelines and on the ClinGen/CGC/VICC standard operating procedure, and a manual review of the literature were used to assist in the classification of variants. After these classification steps, the clinical tiers I, II, and III harboring biologically classified pathogenic or likely pathogenic variants were taken into consideration, while the clinical tier III in the case of a VUS was not retained.

### 4.7. Statistical Analysis

Continuous and categorical data are reported as medians and ranges (min, max) or frequencies and percentages, respectively. The differences between cfDNA concentrations in DFs and plasma and between the patients (overall population and distinct subgroups including malignant or benign diseases) and controls were analyzed by the Wilcoxon signed-rank test. Spearman’s rank correlation coefficient was calculated to evaluate the associations between cfDNA concentrations in plasma and DF samples and between cfDNA concentrations in each body source and the levels of the CA19-9 tumor marker. The data were analyzed by using GraphPad Prism 9.5.1 software. *p* values less than 0.05 were considered to indicate statistical significance. 

## Figures and Tables

**Table 1:** Concentration of cfDNA in duodenal fluid and plasma samples from the study population.

		cfDNA Concentration (ng/mL)	
	Sample ID	Duodenal Fluid	Plasma
IPMN-derived PDAC	#K471	1375	68.25
PDAC	#K476	Not available	41.5
PDAC	#K487	1050	25.5
PDAC	#K506	976.8	97.02
CCA	#K509	151.36	94.6
CCA	#K510	264	77.44
IPMN	#K419	314.6	100.1
IPMN	#K397	627	9.5
Chronic pancreatitis	#K401	598.8	36.96
Chronic pancreatitis	#K402	173.8	91.52
Control	#C399	2500	42
Control	#C406	1092.5	8.8
Control	#C409	2500	11.88
Control	#C410	43.89	34.65
Control	#C411	28.38	30.25
Control	#C416	902.5	285
Control	#C417	250	70.62
Control	#C426	231	98.56
Duodenal Fluid vs. Plasma			
Cases		*p* < 0.0001	
Malignat Cases		*p* = 0.004	
Benign Cases		*p* = 0.029	
Controls		*p* = 0.027	

PDAC: pancreatic ductal adenocarcinoma; CCA: cholangiocarcinoma; IPMN: intraductal papillary mucinous neoplasm.

**Table 2:** Patients with biliopancreatic diseases and controls enrolled into this study.

	PDAC ^§^ N = 4	CCAs N = 2	IPMN N = 2	Pancreatitis N = 2	Controls N = 8
Age, media ± SD	68 ± 16	84 ± 5	75 ± 15	62 ± 8	68 ± 18
≥55 years, n (%)	3 (75)	2 (100)	2 (100)	2 (100)	6 (75)
Gender, N male (%)	2 (50)	2 (100)	0 (0)	1 (50)	3 (37.5)
Body mass index > 30, n (%)	0 (0)	0 (0)	0 (0)	0 (0)	2 (25)
Heavy smokers, n (%)	4 (100)	0 (0)	0 (0)	1 (50)	5 (63)
Heavy drinkers, n (%)	0 (0)	0 (0)	0 (0)	0 (0)	0 (0)
Diabetes, n (%)	2 (50)	1 (50)	2 (100)	2 (100)	4 (50)
Family history of cancer, n (%) *	3 (75)	0 (0)	2 (100)	2 (100)	4 (50)
CA19-9, UI/mL (median, range)	153.9 (2.2–2374)		196 (6.8–1092.6)		5.9 (0.01–30.1)

PDAC: pancreatic ductal adenocarcinoma; CCAs: cholangiocarcinomas; IPMN: intraductal papillary mucinous neoplasm. § The patient with IPMN-derived PDAC is included in this subset. * Variables with some missing data in PDAC.

## Data Availability

The raw data supporting the conclusions of this article will be made available by the authors on request.

## Supplementary Materials

The following supporting information can be downloaded at https://www.mdpi.com/article/10.3390/ijms25158436/s1.
